# Depth-of-interaction encoding techniques for pixelated PET detectors enabled by machine learning methods and fast waveform digitization

**DOI:** 10.1088/1361-6560/adc96d

**Published:** 2025-04-14

**Authors:** Bing Dai, Srilalan Krishnamoorthy, Emmanuel Morales, Suleman Surti, Joel S Karp

**Affiliations:** 1 Department of Radiology, The University of Pennsylvania, Philadelphia, PA 19104, United States of America; 2 Department of Physics and Astronomy, University of Pennsylvania, Philadelphia, PA 19104, United States of America

**Keywords:** depth-of-interaction (DOI), machine learning (ML), long short-term memory (LSTM), waveform sampling (WFS)

## Abstract

*Objective*. Pixelated detectors with single-ended readout are routinely used by commercial positron emission tomography scanners owing to their good energy and timing resolution and optimized manufacturing, but they typically do not provide depth-of-interaction (DOI) information, which can help improve the performance of systems with higher resolution and smaller ring diameter. This work aims to develop a technique for multi-level DOI classification that does not require modifications to the detector designs. *Approach*. We leveraged high-speed (5 Gs s^−1^) waveform sampling electronics with the Domino Ring Sampler (DRS4) and machine learning (ML) methods to extract DOI information from the entire scintillation waveforms of pixelated crystals. We evaluated different grouping schemes for multi-level DOI classification by analyzing the DOI positioning profile and DOI positioning error. We examined trade-offs among crystal configurations, detector timing performance, and DOI classification accuracy. We also investigated the impact of different ML algorithms and input features—extracted from scintillation waveforms—on model accuracy. *Main results*. The DOI positioning profile and positioning error suggest that 2- or 3-level binning was effective for 20 mm long crystals. 2-level discrete DOI models achieved 95% class-wise accuracy and 83% overall accuracy in positioning events into the correct DOI level and 3-level up to 90% class-wise accuracy for long and narrow crystals (2 × 2 × 20 mm^3^). Long short-term memory networks trained with time–frequency moments were twice as efficient in training time while maintaining equal or better accuracy compared to those trained with waveforms. Classical ML algorithms exhibit comparable accuracy while consuming one order less training time than deep learning models. *Significance*. This work demonstrates a proof-of-concept approach for obtaining DOI information from commercially available pixelated detectors without altering the detector design thereby avoiding potential degradation in detector timing performance. It provides an alternative solution for multi-level DOI classification, potentially inspiring future scanner designs.

## 1. Introduction

Commercial positron emission tomography (PET) scanners for human imaging use pixelated detectors with single-ended readout due to their good timing and energy performance and optimized manufacturability (Hsu *et al* 2017, Rausch *et al* 2019, Van Sluis *et al* 2019, Spencer *et al* 2021). Such detectors typically do not provide depth-of-interaction (DOI) information, which helps reduce parallax error, i.e. mis-assignment of line-of-responses for oblique coincidences in 3D PET (Vandenberghe *et al* 2020). Parallax error is normally not a major concern for whole-body PET scanners. However, scanners with smaller ring diameters or using long crystals with small cross-section (such as small animal and organ-specific scanners) suffer from non-uniform spatial resolution within the scanner field-of-view due to increased parallax error and hence could benefit from DOI information.

Unlike monolithic crystals for which DOI information is encoded intrinsically within the scintillation light distribution (Bruyndonckx *et al* 2004, Iborra *et al* 2019, Müller *et al* 2019), modifications to the detector design are usually required for pixelated crystals to obtain DOI information. Beyond dual-ended readout (Shimizu *et al* 1988) that adds cost and complexity to manufacturing, DOI encoding techniques in the literature for pixelated crystals with single-ended readouts can be generally categorized as being either spatial or signal-based methods. As with monolithic detectors, spatial methods introduce DOI dependency in the 2D crystal position map by various light sharing schemes, e.g. offsets between multiple crystal layers (Robar *et al* 1997, Liu *et al* 2001, Ito *et al* 2010, Kuang *et al* 2018, Van Elburg *et al* 2019), depth-dependent depolishing or carvings on crystal sides (Ito *et al* 2011, Pizzichemi *et al* 2016, Nadig *et al* 2023), reflectors between single-layer crystals (Yang *et al* 2009, Ito *et al* 2010, 2013) or between crystal layers (Murayama *et al* 1998, Tsuda *et al* 2004), shared light guide (Pizzichemi *et al* 2016, Zatcepin *et al* 2020, Zeng *et al* 2023), and U-shaped crystals (Li *et al* 2024). While providing DOI capability of 2–3 mm for 20 mm long crystals (Kuang *et al* 2018, Zeng *et al* 2023), some of these designs have some trade-offs, e.g. increased complexity and crystal array manufacturing cost, and in some other cases, degraded light collection efficiency which can result in mis-positioning, loss in energy or timing resolution, with reported coincidence time resolution (CTR) often not matching the performance of some state-of-art commercial PET scanners (Rausch *et al* 2019, Zhang *et al* 2024). The signal-based methods, on the other hand, employ pulse shape discrimination (PSD) to determine the crystal layer of interaction. Berg *et al* (2016) used phosphor coating on a polished crystal to demonstrate DOI encoding with minimal degradation in energy and timing resolution (3% and 9%, respectively). However this method has not been evaluated for its ability to push CTR $ < 200$ ps. Some other works used two crystal layers with different decay time (Schmand *et al* 1998) or rise time (Wiener *et al* 2013, Schmall *et al* 2015) for DOI discrimination. Such methods depend on the statistical quality of the individual signals, and trade-offs need to be considered between the accuracy of DOI discrimination and the timing performance. The increased cost and complexity in system integration also remains a concern.

Most DOI encoding techniques for monolithic detectors (Bruyndonckx *et al* 2004, Iborra *et al* 2019, Müller *et al* 2019) and some for pixelated ones as well (Pizzichemi *et al* 2016, Zatcepin *et al* 2020) estimate continuous DOI positions in length (hence, regression models). Many signal shape methods (Schmand *et al* 1998, Wiener *et al* 2013, Schmall *et al* 2015), on the other hand, employ discrete encoding and classify events to DOI bins (levels). The DOI levels were defined physically by using crystal layers (Schmand *et al* 1998, Wiener *et al* 2013, Schmall *et al* 2015) and multi-level discrimination poses additional challenges to the detector design.

Recently a novel DOI encoding technique has been proposed for single-layer pixelated crystals based on a long short-term memory (LSTM) network trained using energy signals from analog silicon photomultiplier (SiPMs) without any modifications to their hardware (Teimoorisichani *et al* 2023). Dual-level DOI encoding was achieved by grouping discrete measurement depths (layers) to two virtual levels. Measurements based on the detector block from the Biograph Vision which uses an array of $3.2\times3.2\times20~\text{mm}^{3}$ LSO crystal resulted in $ > 80\%$ DOI accuracy at top and bottom layers of the detector and overall accuracy of 76% across all interaction depths with a 2-level classification model. However, the low signal sampling rate (60 MHz) may restrict information that can be extracted from the waveforms, and consequently limit classification accuracy, the choice of machine learning (ML) algorithms, and the ability to investigate different grouping schemes that would extend DOI encoding beyond two levels investigated in that work.

The sampling rate limitation in Teimoorisichani *et al* (2023) can be overcome with high-speed waveform sampling (WFS) electronics such as that provided by the Domino Ring Sampler (DRS4 (Ritt *et al* 2010, Ronzhin *et al* 2013)). The capability of such DRS4-based WFS boards for achieving oscilloscope-quality sampling for large systems has been demonstrated by our group on both a whole-body (WB) time-of-flight (TOF) PET scanner (Ashmanskas *et al* 2014) and a dedicated breast PET scanner (Krishnamoorthy *et al* 2018, 2020).

In this work, we present several alternative ML methods, both traditional and deep learning (DL)-based, for estimating DOI from waveforms digitized at high sample rate with commercially available scintillators and SiPMs. We assess different grouping schemes by computing the width of DOI positioning profile and DOI positioning error. Further, we evaluate these ML-based DOI encoding techniques through tradeoffs among crystal configurations (size and reflector), detector timing performance, and DOI classification, thereby demonstrating that a practical method for multi-level DOI classification with high accuracy can be achieved with current detector designs. The impact of different ML algorithms and input features, which are extracted from waveforms and otherwise unavailable without fast sampling, on model accuracy is also studied.

## 2. Materials and methods

### 2.1. Experiment setup and hardware configurations

**Figure 1.**
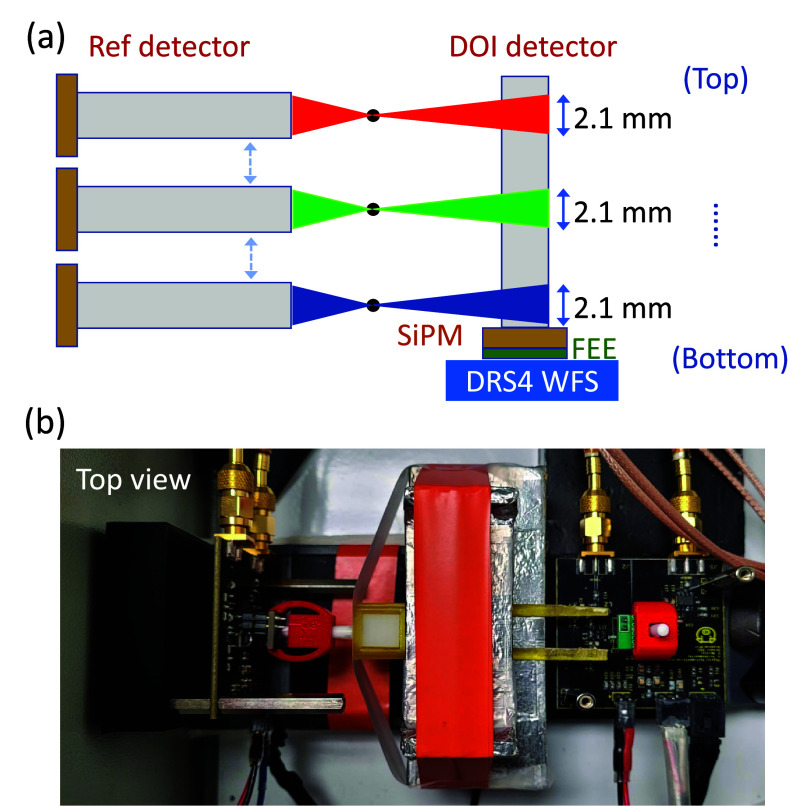
(a) Illustration of side irradiations and (b) top-view picture of the benchtop experimental setup containing the DOI detector with a single crystal and the reference detector. For acquiring ground truth training and test dataset, side irradiations are performed.

As illustrated in figure 1, a long (20 mm) LSO crystal of various configurations as specified in the subsequent paragraph was coupled to a single channel Broadcom SiPM (AFBR-S4N44P014M) in coincidence with a reference detector, which used an identical SiPM but was coupled to a Teflon wrapped $2\times2\times10~\text{mm}^{3}$ LSO crystal. Side irradiation from a 590 kBq ^22^Na point source (0.25 mm activity diameter) at 9 fixed DOI depths created DOI-tagged scintillation events. DOI step of 2.2 mm, slightly larger than the 2.1 mm width of the side irradiation beam, was defined by an additional mechanical collimator (${\sim}2.1$ mm). The size of the beam and the pitch were chosen to achieve good collimation and enough counts for coincidence events in reasonable total acquisition time. For each DOI measurement, the source position was centered at its bin center. This ensured appropriate granularity in DOI measurements and reduced positioning uncertainty that could arise from the use of a beam width larger than the DOI bin size. 5000 energy qualified scintillation events were acquired at each depth, and a single energy window was used. The whole setup operated at room temperature in a light-tight chamber.

**Figure 2.**
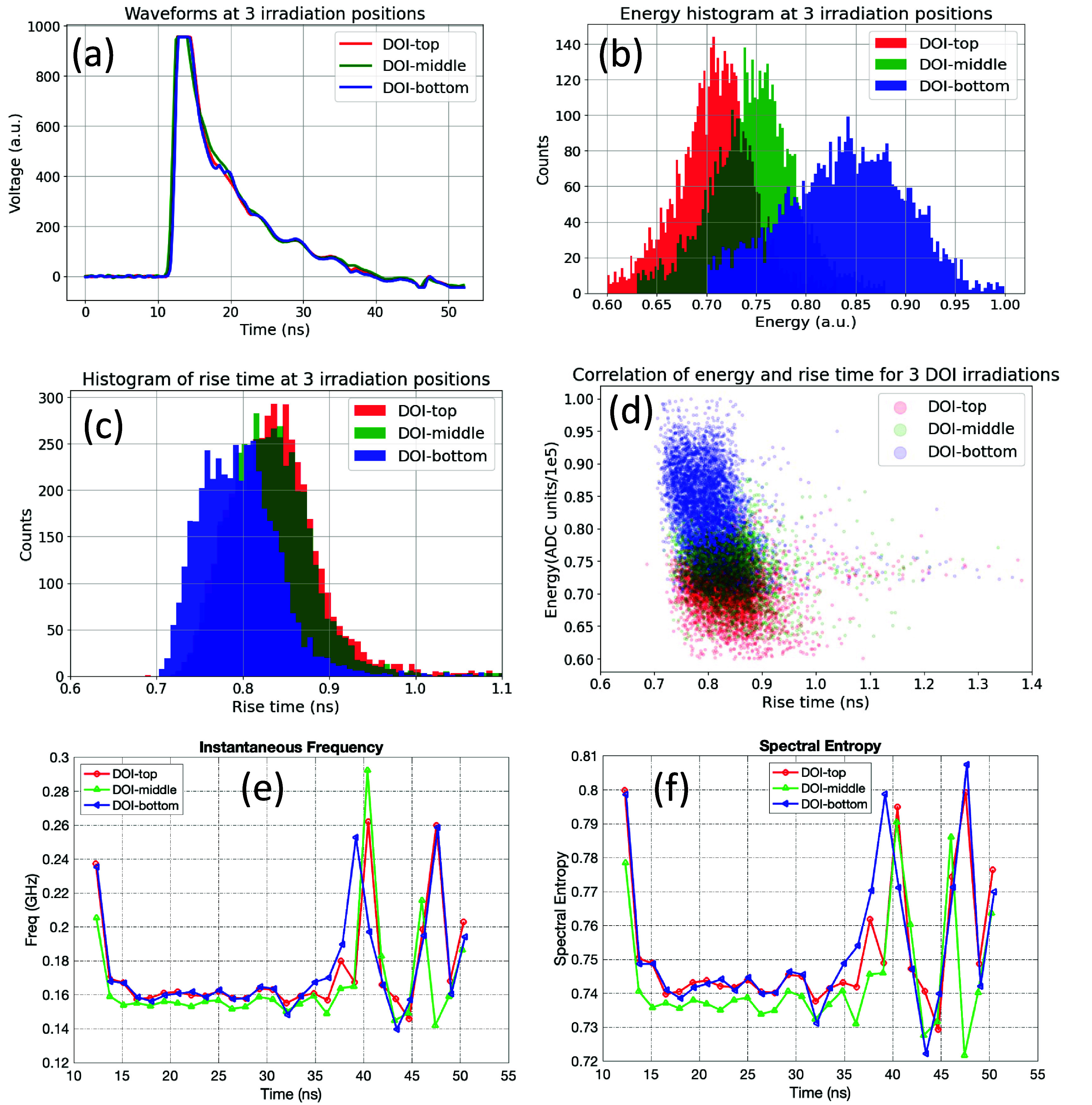
Demonstration of available features from 3-depth DOI measurements (3 side irradiation depths) used for training ML models. (a) Fast timing signal waveforms from one channel at different DOIs. Note that these waveforms were not normalized. (b) Energy histograms, (c) Rise time histograms, (d) Rise time correlation with energy, (e) instantaneous frequency and (f) spectral entropy extracted from the waveforms at different DOIs. Measurements were performed with a $2\times2\times20$ mm^3^ crystal with Teflon wrapping.

Numerous recent studies have shown that low noise and fast front-end electronic (FEE) readout help in achieving the best timing resolution. The FEE developed for this work is based on prior high-frequency FEE (Cates *et al* 2018, Gundacker *et al* 2019). Figure 1(b) shows a photo of the board with the SiPM and a Teflon wrapped $2\times2\times20~\text{mm}^{3}$ LSO crystal coupled to it as the DOI detector. However, instead of sampling the pulse at 20 Gs s^−1^, this work used the DRS4-based WFS data acquisition board to sample entire waveforms at 5 Gs s^−1^. For each SiPM, separate electronic readout channels were used for energy (i.e. total photon counts) and timing signals, and with the timing signal intentionally saturated (at 950 mV) for optimal CTR performance (hence the squared top shown in figure 2(a)).

DOI measurements were performed by varying the following crystal configurations.

(i) *Size of scintillator crystals*: Scintillator crystals of two sizes were measured: $2\times2\times20$ mm^3^ and $3.2\times3.2\times20$ mm^3^.
(ii) *Reflective wrapping*: Each crystal was tested in two ways: (i) with wrapping in highly reflective Teflon tape on five sides, to achieve the highest light output, and (ii) without any reflector wrapping (bare) to better represent air coupling in a crystal array.

The $3.2\times3.2\times20$ mm^3^ crystal, and surface finish for all the above crystals including the reference crystal, are identical to the ones used in the Biograph Vision (Van Sluis *et al* 2019, Teimoorisichani *et al* 2023). The $2\times2\times20$ mm^3^ crystal, to which the same fabrication approach for detector blocks as the $3.2\times3.2\times20$ mm^3^ crystal can be applied, was chosen to investigate the effect of using a higher aspect ratio crystal. The crystals were coupled to the SiPM using optical grease (Saint Gobain BC-630).

After the DOI measurements, the setup was switched to head-on irradiation to characterize the CTR for the each of the above crystal configurations under test. The CTR reported in this paper is the estimated CTR for the two detectors, and is calculated by subtracting in quadrature the timing contribution of the reference detector, and then multiplied by $\sqrt{2}$.

### 2.2. Data preparation (feature extraction)

The fine details of scintillation signals were captured with fast WFS, as evidenced by the distinctions among the shapes of the falling edge of waveforms at different DOIs (figure 2(a)). This capability allows for extraction of numerous features from the waveforms for model training. Rise time (figure 2(c)), 10% to 80% as defined by the saturated signal, was extracted from the rising edge of the waveforms. Further, inspired by prior work in using time–frequency (TF) moments to classify ECG pulses (Kł osowski *et al* 2020), two TF moments (figures 2(e) and (f)) were extracted from the entire waveforms: instantaneous frequency (IF), which represents the rate at which the phase of a signal changes with respect to time and was calculated from time derivative of the phase of Hilbert transformed signal of the original signal (Oppenheim and Schafer 2009), and spectral entropy (SE), which measures the randomness of a signal’s frequency content and was calculated from short-time spectrogram with a rolling time window (width of 12.8 ns with step size of 0.2 ns) (Oppenheim and Schafer 2009). All these features were utilized for training ML models, which include both classical ML and LSTM and are all supervised learning techniques, in the remainder of the paper, unless otherwise specified.

### 2.3. Grouping schemes

A 9-depth measurement was grouped into different number of levels to define DOI bins for classification models. The grouping schemes evaluated in this paper are listed in table 1. In the remainder of the paper, the term ‘*n*-level’ refers to the number of virtual layers in DOI models and ‘*m*-layer’ the number of physical interaction depths in the DOI measurement (where *n* is an integer between 2 and 9 and *m* = 9), unless otherwise specified.

**Table 1.** Grouping schemes for 9-layer DOI measurements. Top layer corresponds to the position (0–2 mm) furthest away from the SiPM and bottom the closest position (18–20 mm).

Grouping scheme	Layer assignment
9-level	one level corresponds to one layer
4-level	9 layers are grouped to 4 levels in 3–2–2–2 arrangement from top to bottom
3-level	9 layers to 3 levels in 3–3–3 arrangement
2-level-54	9 layers to 2 levels in 5–4 arrangement
2-level-45	9 layers to 2 levels in 4–5 arrangement

### 2.4. DOI models—algorithms and input features

The classical ML algorithms adopted in this work include support vector machine, k-nearest neighbors (KNN), random forests, and gradient tree boosting (GTB). Energy (E) and rise time (RT), along with their correlation RT_E (figure 2(d)), were employed as scalar input features for model training. The hyperparameters for each type of classical ML algorithm, such as the number of neighbors for KNN and the number of decision trees for GTB, varied depending on the type of input features. Further details about the classical ML methods are provided in appendix A.

Bi-directional LSTM networks (The Linux Foundation 2023), a type of recurrent neural network, were utilized to learn the DOI dependency from 1D signals. In this work, waveforms (W) and TF features (IF and SE), as well as their combinations, were used as 1D vectors to train LSTM models. Given the distinction in energy histograms for events originating from different irradiation depths (figure 2(b)), energy information (E) was also included. The hyperparameters for LSTM networks were: 100 hidden units, fully connected layer, classification using cross-entropy loss, Adam optimizer, a batch size of 150, a base learning rate of 0.01 with 100 warmup steps followed by a linear decay scheduler (multiplicative factor of 0.5) for every 5 epochs, 50 epochs in total. These settings remained consistent across all LSTM models. The models were built upon the PyTorch framework and ran on Apple Silicon M1 chip with Metal Performance Shaders enabled for GPU training acceleration. More details about the LSTM network architecture are described in appendix B. Note that the hyperparameters are specified as general guidelines rather than optimized values.

The input features for classical ML and LSTM models are listed in table 2. A set of input sequences was created from the measured data according to a grouping scheme and the selected input feature. It was then randomly partitioned into training, validation, and test sets in an $8:1:1$ ratio. A model was trained using grouped data; the trained model was then applied to each layer of the input sequence (ungrouped data) to obtain class-wise accuracy, i.e. the probability of assigning events to the correct DOI bin at each DOI layer. Overall accuracy is defined as the probability of correct prediction for all events across all DOI layers. Each LSTM model was trained for five repetitions to get statistics, and the mean value is used to represent overall accuracy of the model unless otherwise specified, whereas each classical ML model was trained only once, as its accuracy remained consistent once the hyperparameters were fixed.

**Table 2.** List of input features used to train ML models. RT: rise time. E: energy. W: waveform. IF: instantaneous frequency. SE: spectral entropy. Input sequence X_Y corresponds to a combination of feature X and Y. Both IF and SE are time–frequency (TF) features.

	Input features
Classical ML models	RT, E, RT_E
LSTM models	W, IF, SE, IF_SE, W_E, IF_E, SE_E, IF_SE_E

### 2.5. Evaluation of DOI models with different grouping schemes

Model accuracy is not an appropriate metric for evaluating DOI encoding with different grouping schemes, as it depends on bin (level) size—larger bins (less levels) automatically lead to better accuracy. Therefore, two alternative methods, which translate the percentage accuracy of DOI bin classification to predicted DOI value (or error) in mm over all interaction depths (Müller *et al* 2019, Nadig *et al* 2023), were adopted to evaluate the impact of grouping schemes on DOI classification and to identify the most suitable scheme to characterize the DOI positioning for the 9-layer measurements.

(i) *Width of DOI positioning profile*: For each interaction depth, the probability distribution function (PDF) for scintillation events along the crystal length, i.e. the fraction of events in each DOI bin (level), can be estimated by a ML model. As each level represents a distance from the true DOI, the event distribution in crystal length can be approximated as the width (in mm) of the DOI positioning profile within which 68% events fall—this is analogous to $\pm1\sigma$ for a Gaussian distribution. The algorithm to estimate the width of the profile for each DOI layer is to first calculate a cumulative distribution function (CDF) from the PDF based on the trapezoidal rule, then apply a sliding probability window to CDF enclosing the true DOI so that the difference between upper and lower bound of the probability window is 68% while the corresponding width of profile is minimized. The error is estimated by the standard deviation of the profile width over all depths. As the PDF becomes more discrete i.e. with less levels (e.g. two or three levels), resulting in larger fitting error (Nadig *et al* 2023), only the 9-level model trained with ungrouped data was used for this method.
(ii) *DOI positioning error*: DOI positioning error is defined as the absolute distance between the predicted DOI and the true DOI, which is the known interaction depth. Positioning error for DOI classification is attributed to two factors. (1) DOI binning: grouping several DOI layers to one level leads to displacement between the interaction depths (true DOI) and the center of the DOI level (designated DOI). (2) Model prediction: for each DOI layer, assignment of scintillation events to *n* DOI levels follows a probability distribution predicted by the *n*-level model. False predictions lead to positioning error. Together, DOI positioning error for each DOI layer with a given grouping scheme can be calculated by weighting the displacement due to DOI binning with the probability distribution from model prediction across all DOI levels. Unlike the first method, this method is less affected by data sparsity associated with the number of DOI levels (Müller *et al* 2019, Nadig *et al* 2023) and thus not limited to ungrouped data. As the positioning error treats positive and negative displacement the same, the mean error is expected to be about the half of the width of DOI positioning profile.

The $2\times2\times20~\text{mm}^3$ crystal with Teflon wrapping was selected to evaluate grouping schemes for two reasons: (1) The smaller cross-section of the crystal provides better spatial resolution, making it particularly relevant for high-performance scanners; (2) Teflon wrapping improves light collection and mitigates the influence of varying photon collection as a function of DOI on the evaluation.

### 2.6. Evaluation of the tradeoff among crystal configurations, detector timing performance, and doi classification

Once the most suitable grouping scheme is determined using the profile width and positioning error, we then select the highest overall accuracy from the models trained with different input features to represent model accuracy and use it as the metric to evaluate the tradeoffs among crystal configurations, detector timing performance, and DOI classification. The hyperparameters were fixed across all models with different crystal configurations and input features to ensure consistency in comparing model accuracies. Because the input features for classical ML models and LSTM models differ (table 2), we treated the two types of models separately, resulting in two DOI accuracies for each crystal configuration—one for classical ML and one for LSTM models. In the remainder of the paper, the term ‘ML’ in the legends of all figures refers to classical ML algorithms, unless otherwise specified.

Using the metric, we identified the crystal configuration that resulted in high (though not necessarily optimal) model accuracy with good detector timing performance. We then reported the best models (for LSTM models, it is the one with the highest overall accuracy out of five repetitions) for such configuration.

### 2.7. Evaluation of different ML algorithms and input features

With the selected grouping scheme, we also investigated how different ML algorithms and input features affect model accuracy. Specifically, we compared: (1) LSTM models versus classical ML models, (2) models trained with versus without energy information, (3) models trained with waveforms versus those trained with TF features, and (4) classical ML models trained with RT, energy, and energy-RT correlation and note that identifying the ‘best’ input feature (e.g. SE versus IF) or ML algorithm (e.g. RF versus GTB) is beyond the scope of this work.

## 3. Results

### 3.1. DOI classification with different grouping schemes

**Figure 3.**
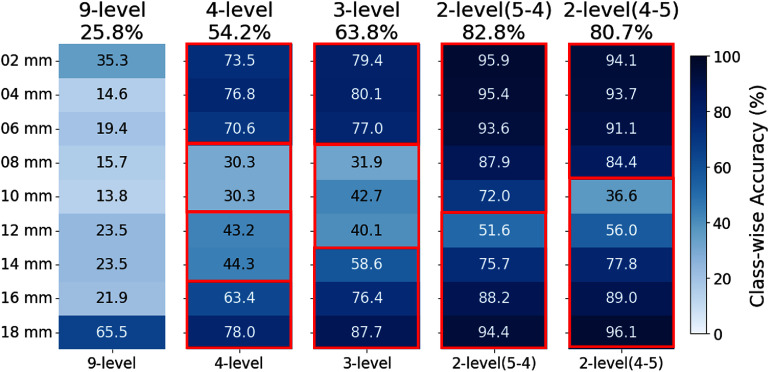
Class-wise accuracies (labeled inside the heatmaps) for different grouping schemes labeled by red boundaries. Overall accuracy is listed above the heatmaps. All models were LSTM networks trained with the same waveforms along with energy information (W_E), measured on a $2\times2\times20$ mm^3^ crystal with Teflon wrapping.

**Figure 4.**
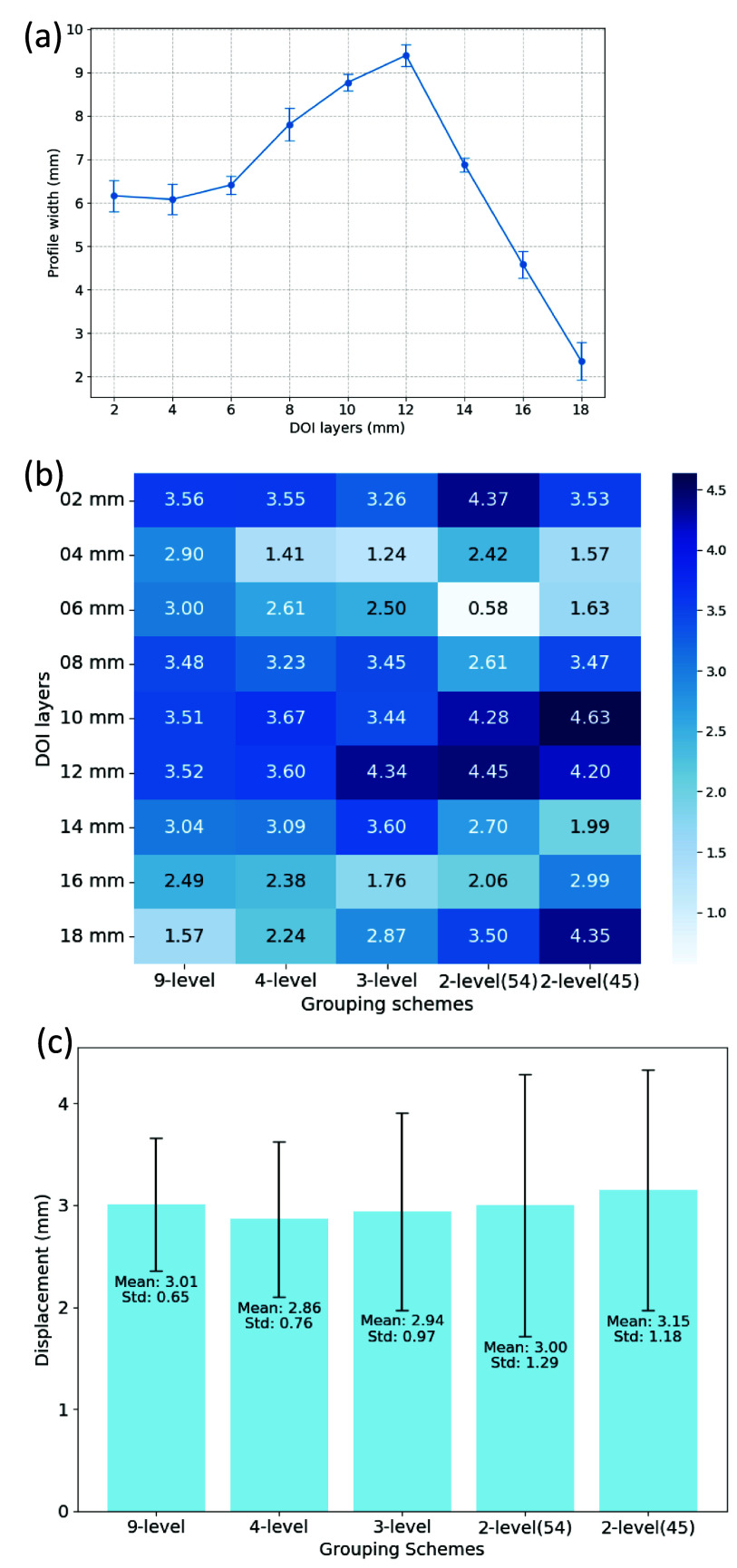
(a) Width of DOI positioning profile at 9 DOI layers, calculated from the same 9-level LSTM model used in figure 3. (b) DOI positioning error at 9 DOI layers, and (c) its mean and std error over all depths for different grouping schemes, calculated from corresponding 9-level to 2-level LSTM models used in figure 3.

Figure 3 shows the overall accuracy and class-wise accuracies of 9-level, 4-level, 3-level, and 2-level LSTM models trained with the same waveforms and energy information from measurements performed on a $2\times2\times20~\text{mm}^3$ crystal with Teflon wrapping. The overall accuracy increases with less DOI levels, as expected. The class-wise accuracies are lower at the boundaries of DOI levels, agreeing with the increased width of DOI positioning profile (figure 4(a)) and positioning error (figure 4(b)) in the middle of the crystal (DOI layers 6–14 mm). The profile width is $ > 6$ mm for DOI layers 6–14 mm (figure 4(a)). Correspondingly, the positioning error is mostly $ > 3$ mm for the same 6–14 mm layers (figure 4(b)). The 2 × relationship between the profile width and the positioning error agrees with the way they are calculated, as described in section 2.5. The mean width of profile over all depths is $6.5 (\pm2.1)$ mm, indicating that either 3-level or 2-level grouping scheme is sufficient for 9-layer measurements on a 20 mm long crystal, and that DOI encoding will help reduce the parallax error of a long crystal to 1/3–1/2 of the crystal length. On the other hand, the mean positioning error (figure 4(c)) is similar (${\sim}3$ mm) across different grouping schemes, although it is marginally higher with 2-level-45 and fluctuates more (i.e. larger std error) with less DOI levels along crystal length. Considering both metrics, the 3-level scheme was selected for the evaluations in the next sections. Note that the tick labels ‘02 mm’, ‘04 mm’, etc do not correspond to the actual bin centers but are instead used to denote DOI layers in all figures throughout this paper, unless otherwise specified.

### 3.2. Impact of crystal size and reflector on detector timing performance and model accuracy

**Figure 5.**
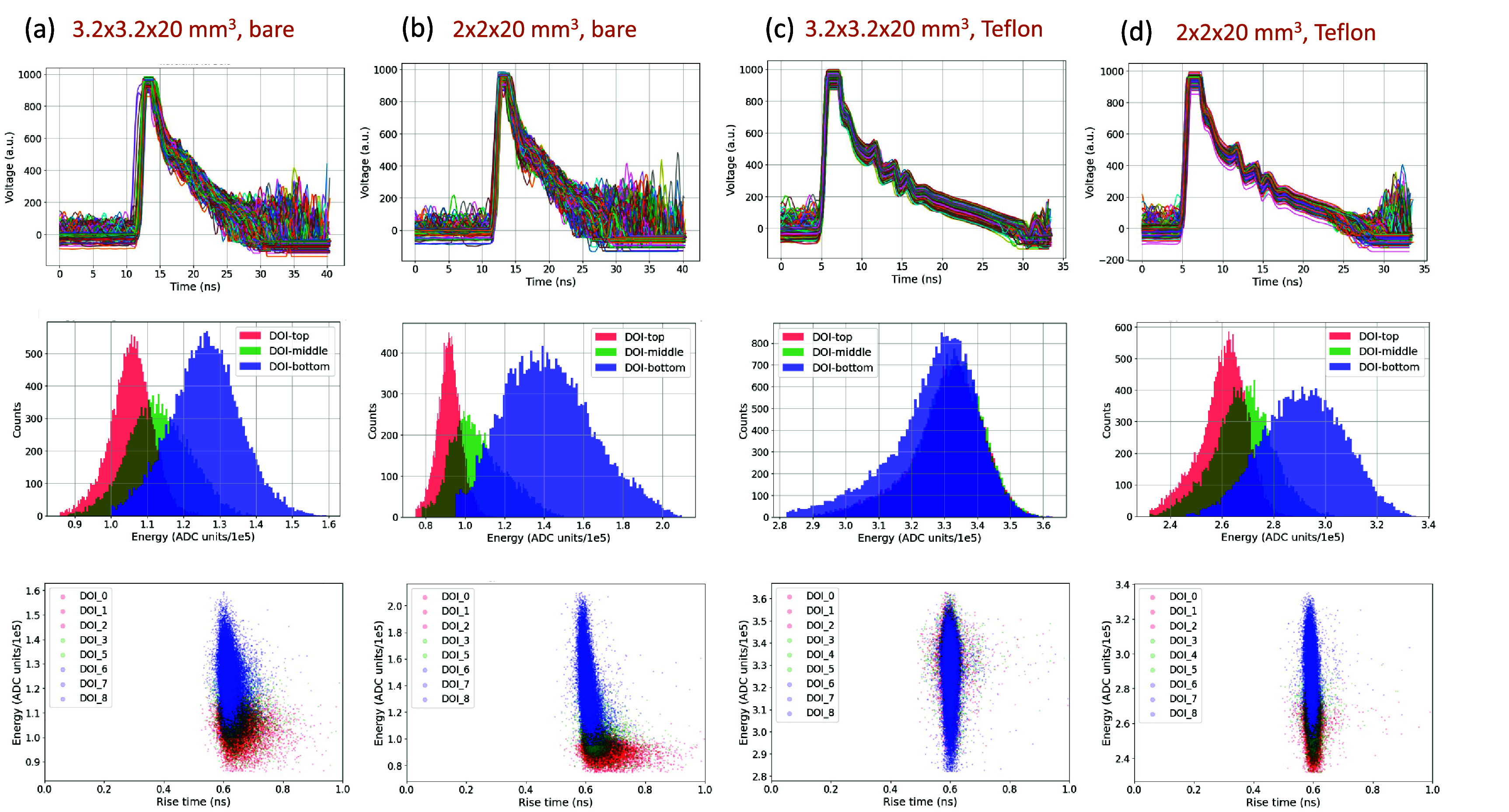
The measured timing waveforms from the top DOI layer (1st row), energy histogram (2nd row), and energy-rise time correlation (3rd row) using the 3-level grouping scheme for (a) $3.2\times3.2\times20~\text{mm}^3$ bare crystal, (b) $2\times2\times20~\text{mm}^3$ bare crystal, (c) $3.2\times3.2\times20~\text{mm}^3$ crystal with Teflon wrapping, and (d) $2\times2\times20~\text{mm}^3$ crystal with Teflon wrapping. The same scale was used for energy measurements of different samples..

**Figure 6.**
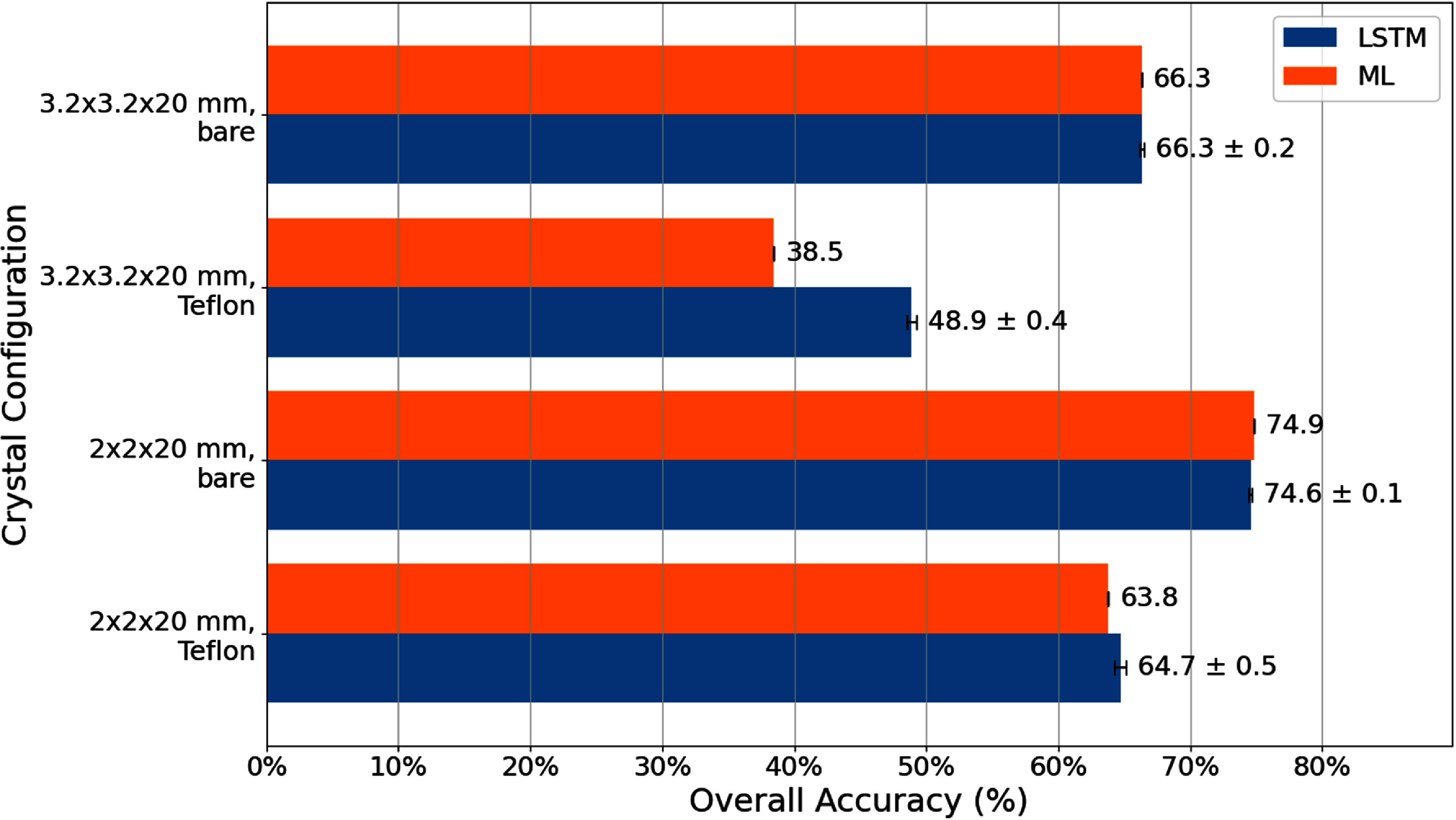
Overall accuracy of classical ML and LSTM models for 3-level DOI models with different crystal configurations.

As indicated by differences in measured timing waveforms, energy histograms, and RTs (figure 5), crystal size and reflector impact detector performance and DOI model accuracy (figure 6).

(i) *Crystal size*: Narrower crystals ($2\times2\times20~\text{mm}^3$) exhibit better DOI accuracy than wider ones ($3.2\times3.2\times20~\text{mm}^3$), 64.7% vs 48.9% with Teflon and 74.9% vs 66.3% without, respectively, while achieving similar CTR ($162\pm5$ ps, with Teflon wrapping) to that of wider ones ($160\pm5$ ps, with Teflon wrapping), despite some loss in light collection from the smaller cross-section.
(ii) *Crystal wrapping*: The bare crystals of both sizes demonstrated better DOI accuracy compared to those with Teflon wrapping, 66.3% vs 48.9% and 74.9% vs 64.7% for $3.2\times3.2\times20~\text{mm}^3$ and $2\times2\times20~\text{mm}^3$ crystals, respectively, although this came at the expense of degraded CTR ($200\pm6$ ps vs $160\pm5$ ps and $190\pm6$ ps vs $162\pm5$ ps, for $3.2\times3.2\times20~\text{mm}^3$ and $2\times2\times20~\text{mm}^3$ crystals, respectively) due to reduced light collection without a reflector.

**Figure 7.**
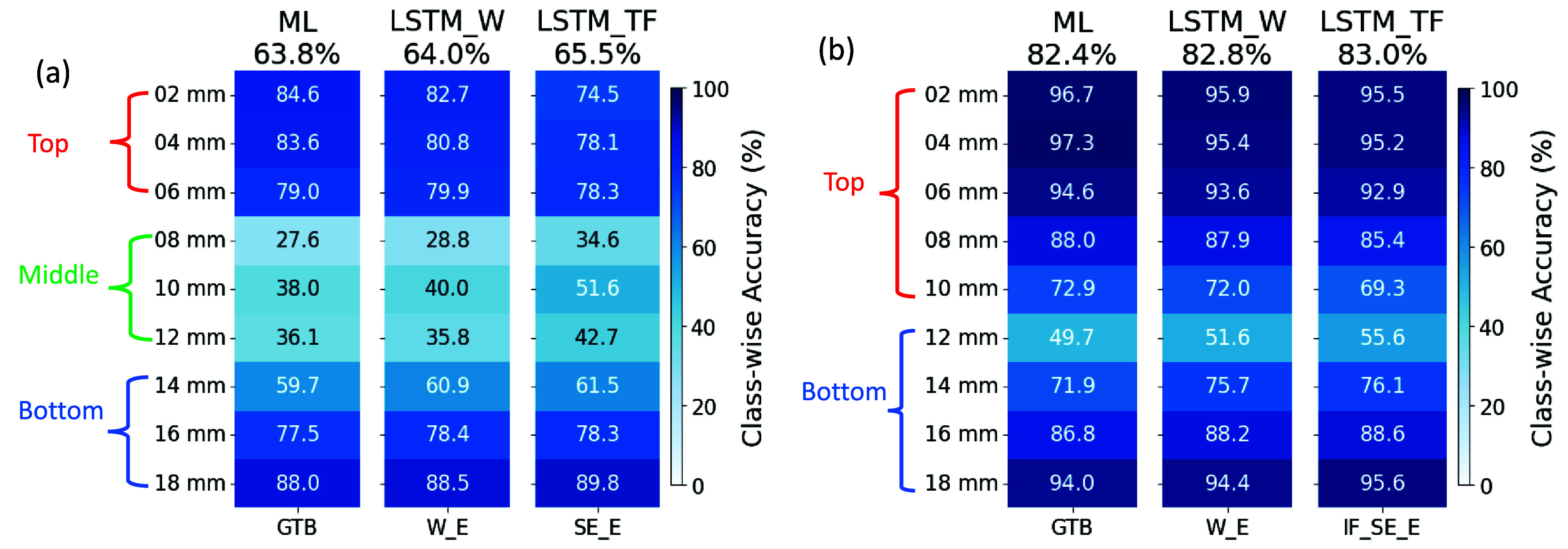
(a) The best 3-level and (b) 2-level (using 2-level-54 scheme) DOI models, trained with different machine learning algorithms and input features using measurements on a $2\times2\times20~\text{mm}^3$ crystal with Teflon wrapping.

Overall, the $2\times2\times20~\text{mm}^3$ crystal with Teflon wrapping provides the best tradeoff between detector timing performance and model accuracy. The optimal 3-level and 2-level DOI models based on this detector configuration are shown in figure 7. The overall accuracy for 2-level DOI discrimination reaches 83% for LSTM models trained with either waveforms (W, specifically W_E) or TF features (TF, specifically IF_SE_E). Class-wise accuracies are 95% at top and bottom layers and drop to 55% at the boundary between top and bottom levels. The overall accuracy for 3-level discrimination is up to 65.5% for LSTM models trained with TF features. The class-wise accuracies are still high at top and bottom layers, up to ∼ 85% (for the ML model trained with GTB) and 90% (for the LSTM model trained with SE_E), respectively. For both 2-level and 3-level DOI discrimination, the differences in accuracy are minor between models trained with different ML algorithms and input features.

### 3.3. Impact of ML algorithms and input features on DOI model accuracy

**Figure 8.**
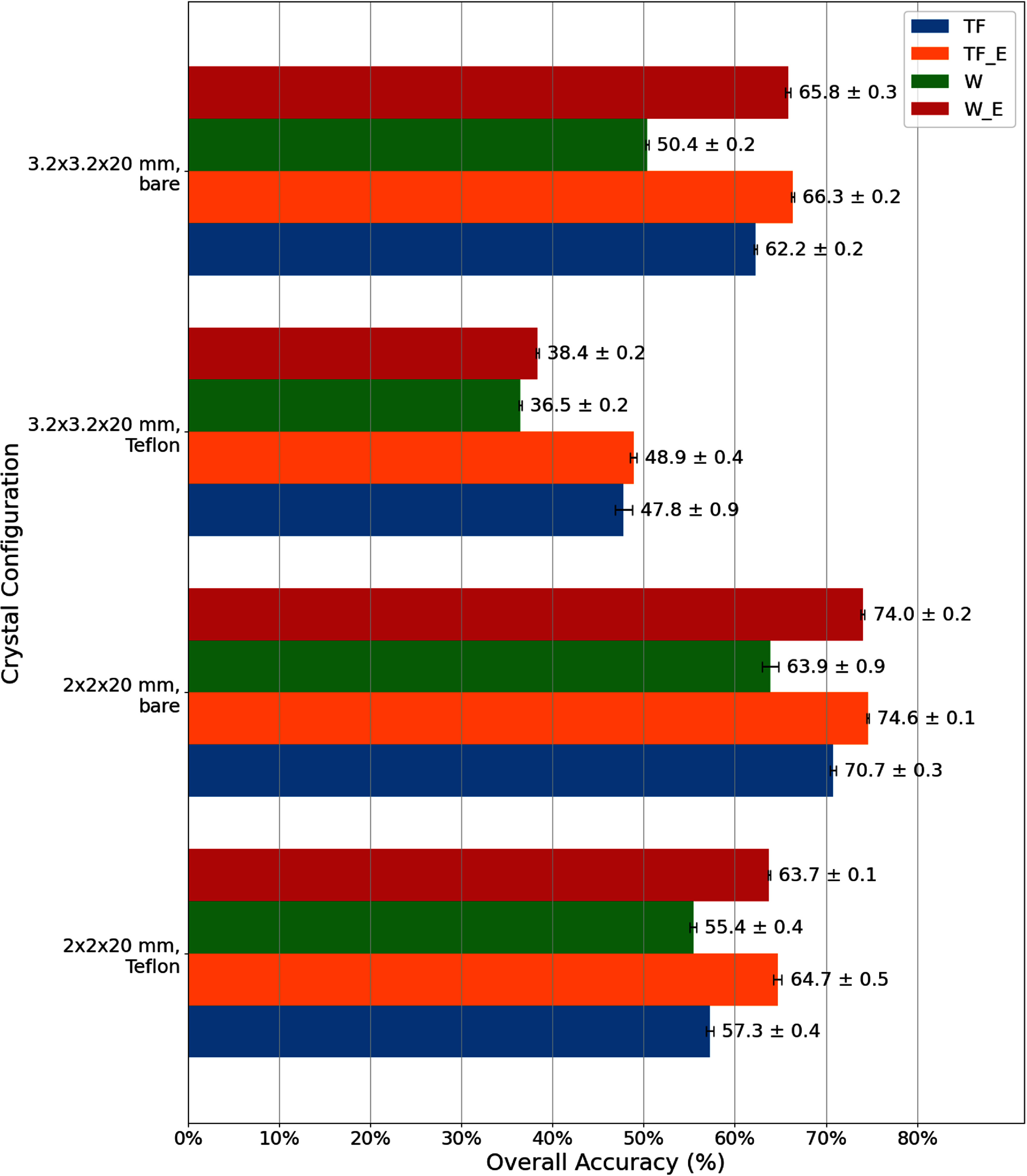
Overall accuracies of 3-level LSTM models, trained using different input features.TF_E: time–frequency features with energy information, W_E: waveforms with energy information, as defined in section 2.2 and table 2.

**Figure 9.**
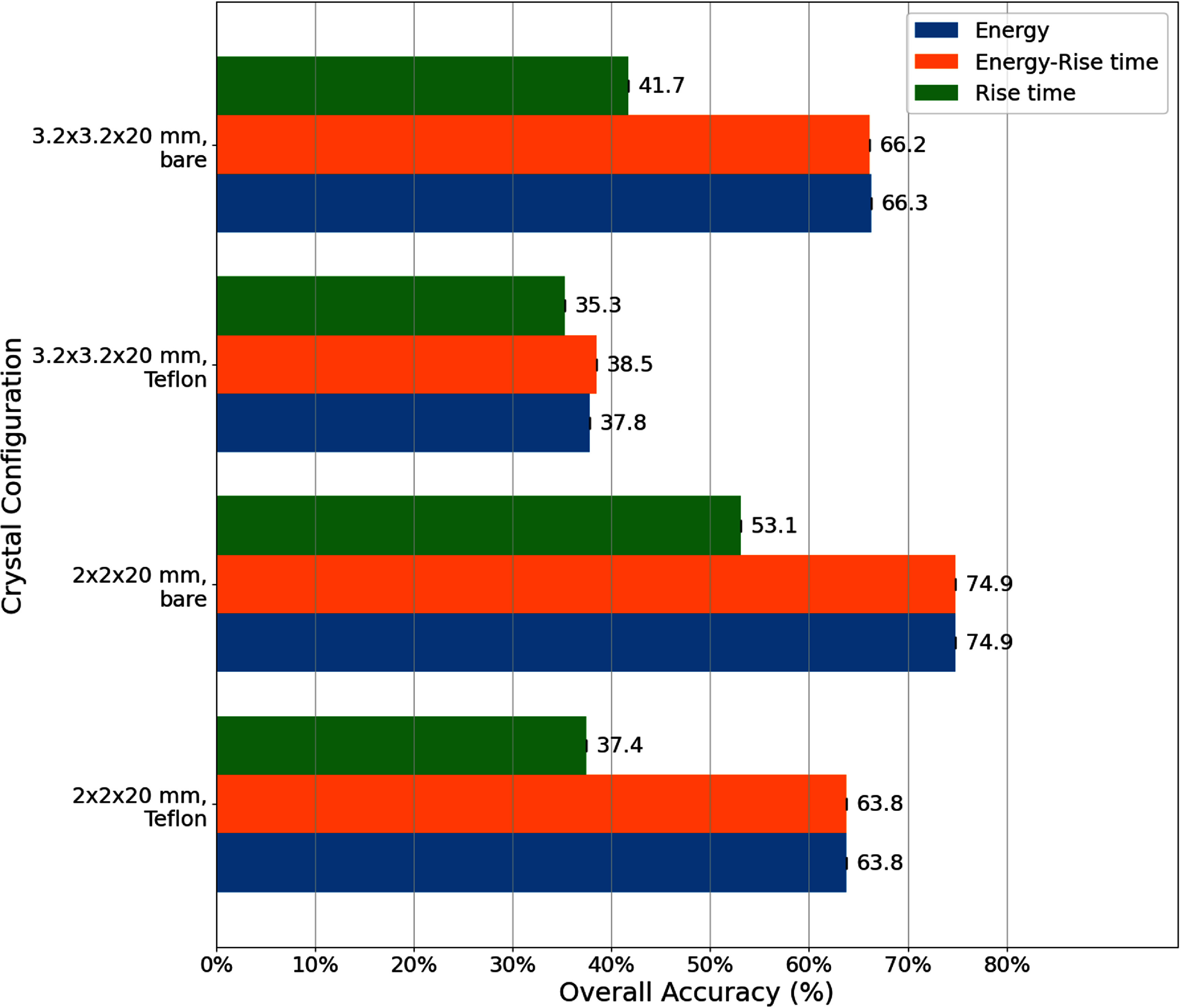
Overall accuracy of 3-level classical ML models for DOI discrimination, trained using rise time, energy, and energy-rise time correlation.

(i) *LSTM models vs classical ML models*: As shown in figure 6, classical ML models overall exhibit comparable accuracy to LSTM models, except for the $3.2\times3.2\times20~\text{mm}^3$ crystal with Teflon wrapping, possibly due to indistinct separation of both energy and RT histograms at different DOI levels (3rd column, figure 5) that classical ML algorithms rely on.
(ii) *Energy information (total photon counts) for model accuracy*: As shown in figure 8, including energy information in conjunction with other input features (both waveforms and TF features) helps improve model accuracy for LSTM models across all crystal configurations. For example, the overall accuracy of the LSTM model for the $3.2\times3.2\times20~\text{mm}^3$ bare crystal was 50.4% when trained with waveforms only (i.e. input feature was W and input_size = 1 for the network), and it increased to 65.8% when energy was added as another input feature to the network (i.e. input features were W_E and input_size = 2). In general, more input features can help the model differentiate between classes more effectively, especially in classification tasks where the original features may not fully capture the distinctions. More discussion will be given in section 4.2.
(iii) *Waveforms and TF features for model accuracy*: As shown in figure 8, LSTM models trained with TF features generally achieve equal or better accuracy compared to those trained with waveforms, regardless of whether energy information is included. In particular, they exhibit better accuracy in the absence of energy information or when energy histograms from different DOIs mostly overlap, e.g. $3.2\times3.2\times20~\text{mm}^3$ with Teflon wrapping (figure 5).
(iv) *RT, energy, and energy-RT correlation for classical ML models*: As evident from figure 9, for all crystal configurations, classical ML models trained with RT alone exhibit lower accuracy compared to those with energy-RT or energy alone, indicating that RT is not as effective as other input features in this work. Meanwhile, models trained with energy-RT demonstrate similar accuracy as those trained with energy alone.

## 4. Discussion

### 4.1. Grouping schemes and model accuracy

The proof-of-concept measurements show that the proposed DOI encoding methods using different grouping schemes do not make a significant difference in the mean DOI positioning error despite the distinct difference in overall modal accuracy. Nevertheless, it is still meaningful because the mean positioning error (3 mm, figure 4(b)) with DOI is better than no DOI where all events are placed at the center of the crystal—5 mm of mean error. Note that when calculating mean positioning error over all DOI layers, equal probability of interaction was assumed in each layer with the side-irradiation measurements. A more realistic way—considering head-on irradiation—would be to weigh the error with the depth-dependent relative probability of interaction. This will be part of our future work.

Model accuracies vary more significantly with different DOI levels (figure 3) than with different input features or ML algorithms (figures 8 and 9). Therefore, the conclusion drawn from one crystal configuration regarding the most suitable grouping scheme, namely, the 3-level or 2-level DOI model for 9-layer measurements, should be applicable to other configurations as well.

The accuracies of our 2-level DOI models (figure 7(b)) using measurements on a $2\times2\times20~\text{mm}^3$ crystal with Teflon wrapping are higher than those reported in literature (Teimoorisichani *et al* 2023), in terms of both average accuracy (83% vs 76%) and class-wise accuracies (up to 96% vs up to 91%). And the class-wise accuracies of our 3-level models (figure 7(a)) are up to 90%, close to those of the 2-level models in literature Teimoorisichani *et al* (2023), indicating that the advantage of increased WFS frequency becomes more significant with more DOI levels. Admittedly, the comparison above may not be entirely fair because of differences in crystal configurations, experimental setup, algorithms, etc. Nevertheless, this result suggests that our proposed method may be more advantageous in case multi-level DOI discrimination is demanded.

### 4.2. Energy information and separation in energy histograms

As described in section 3.3, energy information plays an important role in DOI classification in this work, namely, incorporating energy information improves model accuracy. We further observed, as shown in figures 5, 6 and 8, a strong correlation between high model accuracy and clear distinction in energy histograms between scintillation events from different DOI levels. This is evident when comparing the energy histograms and model accuracy of $3.2\times3.2\times20~\text{mm}^3$ crystal with Teflon wrapping to the other three configurations.

On the other hand, even without energy separation, almost 50% overall accuracy for 3-level DOI classification is still achieved with LSTM models for the $3.2\times3.2\times20~\text{mm}^3$ crystal with Teflon wrapping, likely because LSTM performs good DOI discrimination from not only depth-dependent energy histograms but also waveforms and TF features—more will be discussed later in 4.4.

The separation of energy histograms at different DOIs varies with different crystal configurations (the second row in figure 5). Conversely, the DOI dependent variation of energy histograms may lead to the broadening of the energy spectra, evident for the $3.2\times3.2\times20~\text{mm}^3$ crystal with and without Teflon wrapping (figures 5 and 6). For non-DOI detectors, such broadening is not preferred, and reflectors are often used to minimize this effect. For DOI detectors, the tradeoff among reflectors, detector performance, and DOI accuracy should be considered, as discussed in section 3.2.

### 4.3. Rise time

The ineffectiveness of RT for training classical ML models may be due to the difficulty in distinguishing subtle variations in RT for different DOI levels (figure 5), which probably requires signals of statistically higher quality than what is achievable with the WFS setup used in this paper. Consequently, using RT for ML training appears to face similar limitations as using the rising edge in the PSD method for DOI encoding.

On the other hand, reducing the sampling rate should not affect model accuracies for classical ML models when trained with energy information alone. This is worth further investigation for systems with lower sampling rate.

### 4.4. Waveforms and TF features for LSTM models

LSTM models trained with TF features overall outperform those trained with waveforms likely because LSTM networks are more efficient at learning DOI dependencies from TF features than from raw signals, as TF moments can potentially reveal or amplify some DOI-dependent signatures of the signals in the frequency domain that might be otherwise overshadowed by noises in the spatial domain. As demonstrated in figure 2, the raw waveforms—not normalized—at three depths (figure 2(a)) do not appear to differ significantly in shape or size except for some subtle variations in the ripple of the falling edge, while the TF features extracted from them exhibit more distinct differences (figures 2(e) and (f)).

Also note that TF features have shorter sequence lengths than raw waveforms, depending on the size of time window and the step size. Shorter sequences result in faster computation time in training. In this work, LSTM models trained with TF features typically required half the training time compared to those trained with raw waveforms.

Future work could explore additional TF features and conduct theoretical and systematic studies on comparing TF moments with raw waveforms as input features to LSTM networks, or other DL networks designed to handle long short-term dependencies in data sequences.

We compared TF features and raw waveforms as two broad categories of input features for LSTM models, representing the spatial and frequency domains, respectively. However, we did not evaluate specific input features in finer detail, such as individual TF features like IF versus SE versus IF_SE. This is because model accuracy with a particular input feature is influenced by numerous factors—including feature extraction settings, hyperparameters, grouping schemes, and crystal configurations—making it challenging to draw general conclusions at such a detailed level. This reasoning also applies to why identifying the ‘best’ ML algorithms (e.g. RF versus GTB) is beyond the scope of this study.

### 4.5. Comparison of ML Methods

Classical ML algorithms in most cases exhibit accuracy comparable to that of LSTM models, provided the separation between energy histograms for different DOI levels is sufficiently distinct (figures 5 and 6). They often have simpler structures, requiring fewer parameters to tune and less memory, which makes them faster to converge especially on smaller datasets (Goodfellow *et al* 2016). In our work, LSTM models typically took about 5 min to train while classical ML algorithms less than 30 s—an order of magnitude faster in training time. Additionally, these models are well-suited for implementation on field-programmable gate arrays (Müller *et al* 2018, Alcolea and Resano 2021), enabling real-time on-board DOI discrimination.

### 4.6. Detector performance and scalability

We focused on DOI encoding techniques for pixelated detectors with single-ended readout because of their good spatial, energy, and timing resolution as well as manufacturability. However, due to differences in crystal configurations, photon detection efficiency of various SiPMs or between SiPMs and PMTs, readout electronics, etc it is challenging to make a fair comparison of detector performance across different DOI methods in literature. Especially, the methodology proposed in this work, based on WFS and ML, to our knowledge, is the first of its kind and thus there are no prior results available on detector performance for direct comparison.

It is worth noting that the primary aim of the work is to present an alternative approach and a general methodology, via proof-of-concept experimental measurements, that can achieve effective DOI accuracy without modifying the detector design. Measurements in this study are limited to single crystals of two cross sections (2.0 mm and 3.2 mm; with and without Teflon wrapping), as the current FEE only supports single channel readout. Nevertheless, we believe that crystals of such sizes represent a reasonable range of scanners and applications, and bare crystal and Teflon wrapping cover the two extremes of the wide spectrum of numerous reflective properties. As a next step, we will develop a multichannel FEE to readout a $4\times4$ Broadcom SiPM array with technical performance identical to the SiPMs used in this work and will measure crystal arrays. This would help us understand any potential trade-offs between DOI accuracy and detector performance, such as light output variation within an array, upon scaling this technique to a full detector. We do not expect any further performance deterioration when integrating such a detector module into a complete scanner.

The training time scales approximately linearly with input data volume for a fixed configuration. While larger datasets introduce challenges such as data storage and computational overhead, these are comparable to the challenges faced by high-resolution or TB-PET scanners. With modern advancements in distributed computing, high-performance storage solutions, and scalable infrastructure, these issues can be effectively managed and should not be a major concern when scaling up the design. Prior work with similar-sized crystals has suggested that using even a 2-layer DOI for 20 mm long crystals can improve both spatial resolution and reconstructed image quality (Teimoorisichani *et al* 2023).

It is also important to highlight that the high-speed sampling uses DRS4, which, unlike oscilloscopes, can be scaled. In fact, we have developed two DRS4-based WFS data acquisition systems in our previous work—one for a WB TOF PET scanner (Ashmanskas *et al* 2014) and another for a dedicated breast PET scanner (Krishnamoorthy *et al* 2018, 2020), demonstrating our ability to develop scalable electronics using the DRS4 chip. Thus, these proof-of-concept measurements are scalable at the sampling frequency offered by the DRS4 chip. Although the cost of DRS4 chips might be a slight concern, it is not prohibitive for real scanners and is feasible to implement on a scale, unlike some other methods which use $ > 10$ Gsps.

## 5. Conclusion

We demonstrated that fast digitization using WFS on entire scintillation signals enables multi-level DOI classification from waveforms with both LSTM and classical ML methods, without necessitating modifications to the detector designs and thus avoiding potential degradation in CTR that may result from pixelated detector designs intended to obtain the DOI information. Multi-level DOI classification with different grouping schemes was investigated and no significant difference in the mean DOI positioning error was observed from 2 levels to 9 levels. The estimated width of DOI positioning profile suggests that 2- or 3-level binning for 9-depth DOI measurements was appropriate for 20 mm long crystals. Narrower crystals result in high DOI accuracy without compromising CTR. For long and narrow crystals ($2\times2\times20~\text{mm}^3$), 83% overall accuracy and 95% accuracy for top and bottom DOI layers were achieved with 2-level models, and a class-wise accuracy up to 90% with 3-level models, demonstrating the feasibility of the proposed method for accurate DOI classification beyond two levels. Various features can be extracted from waveforms for training DOI models owing to fast digitization. In general, training LSTM models with TF features is more efficient than using raw waveforms, while still attaining better DOI accuracy. Classical ML algorithms takes much less training time while exhibiting comparable accuracy. Also, it is evident that the separation of energy histograms for events from different DOI levels improves model accuracy.

## Figures and Tables

**Figure A1.**
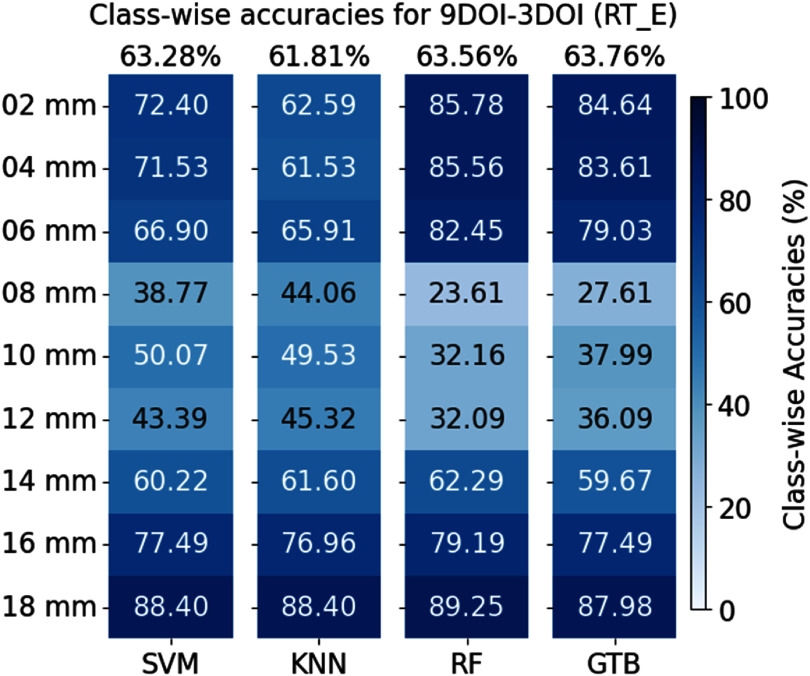
Overall and class-wise accuracies of classical ML models for 3-level DOI classification using 9-depth measurements on a $2\times2\times20~\text{mm}^3$ crystal with Teflon wrapping. The same measurements were used in figure 7(a). The input features were RT_E.

**Figure B1.**
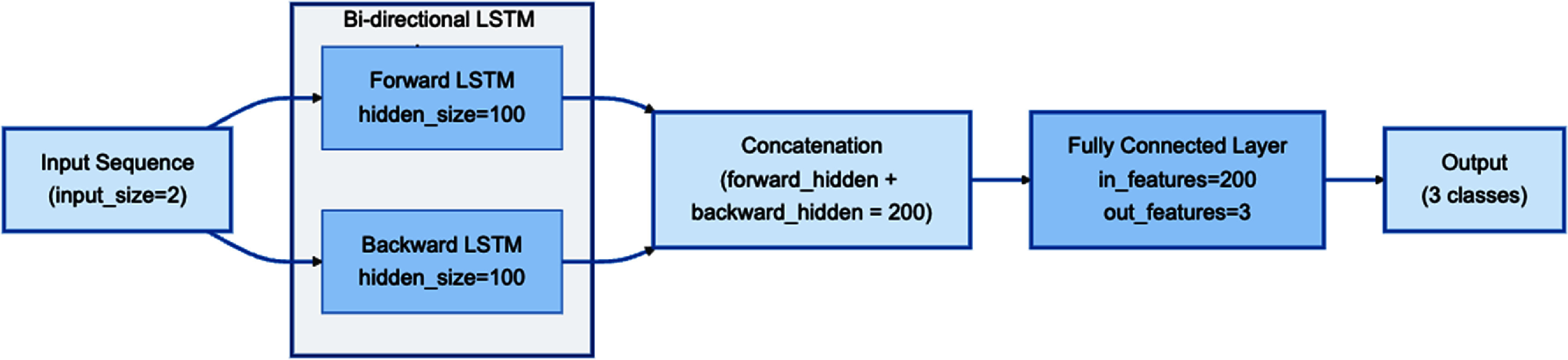
Illustration of a bi-directional LSTM network with hyperparameters depicted in section 2.4. intput_size: the number of input features. out_features: the number of output classes. Both forward and backward LSTMs process the input, each producing *N*-dimensional output where *N* is defined by hidden_size. Output from forward and backward LSTMs are concatenated to form a 2*N*-dimension vector. Softmax is applied across output classes.

## Data Availability

The data cannot be made publicly available upon publication because they are not available in a format that is sufficiently accessible or reusable by other researchers. The data that support the findings of this study are available upon reasonable request from the authors.

## Appendix A Classical ML models

GTB overall performs best with RF being the close second among all classical ML methods investigated in this work. The hyperparameters for a GTB model for 3-level DOI classification using RT_E as input features (figure A1) are listed in table A1.

**Table A1.** Key hyperparameters for a GTB model for 3-level DOI classification. Measurements and input features were the same as those in figure A1.

Key parameter	Value
loss	‘Log_loss’
learning rate	0.1
n_estimator	50
criterion	‘friedman_mse’
min_samples_split	2
min_samples_leaf	1
max_depth	3
max_features	‘sqrt’

## Appendix B LSTM networks

Figure B1 illustrates the bi-directional LSTM network for a 3-level DOI model trained with 2 input features (e.g. W_E) with hyperparameters depicted in section 2.4. Specifically, for each input timestep:

(i) Input has 2 features (input_size = 2)
(ii) Both forward and backward LSTMs process this input, each producing 100-dimensional output (hidden_size = 100)
(iii) These are concatenated to form a 200-dimensional vector (hidden_size ×2)
(iv) The fully connected layer transforms this to 3 output features (out_features = 3)
(v) Softmax transforms the raw outputs of the fully connected layer into probabilities for multi-class classification (e.g. predicting one of 3 classes in this example).
